# Development of a novel biodegradable porous iron-based implant for bone replacement

**DOI:** 10.1038/s41598-020-66289-y

**Published:** 2020-06-04

**Authors:** Bernd Wegener, Anton Sichler, Stefan Milz, Christoph Sprecher, Korbinian Pieper, Walter Hermanns, Volkmar Jansson, Berthold Nies, Bernd Kieback, Peter Ernst Müller, Veronika Wegener, Peter Quadbeck

**Affiliations:** 1 Department of Orthopedic Surgery, Physical Medicine and Rehabilitation, Ludwig-Maximilians-University of Munich, Munich, Germany; 2 Department of Anatomy, Ludwig-Maximilians-University of Munich, Munich, Germany; 3 AO Research Institute, AO Foundation, Davos, Switzerland; 4 Department of Surgery and Genecology of Animals, Ludwig-Maximilians-University Munich, Munich, Germany; 5 Department of Veterinery Pathology, Ludwig-Maximilians-University of Munich, Munich, Germany; 6 InnoTERE GmbH, Dresden, Germany; 7 Fraunhofer Institute for Manufacturing and Advanced Materials (IFAM), Dresden, Germany

**Keywords:** Experimental models of disease, Preclinical research

## Abstract

Bone replacement and osteosynthesis require materials which can at least temporarily bear high mechanical loads. Ideally, these materials would eventually degrade and would be replaced by bone deposited from the host organism. To date several metals, notably iron and iron-based alloys have been identified as suitable materials because they combine high strength at medium corrosion rates. However, currently, these materials do not degrade within an appropriate amount of time. Therefore, the aim of the present study is the development of an iron-based degradable sponge-like (i.e. cellular) implant for bone replacement with biomechanically tailored properties. We used a metal powder sintering approach to manufacture a cylindrical cellular implant which in addition contains phosphor as an alloying element. No corrosion inhibiting effects of phosphorus have been found, the degradation rate was not altered. Implant prototypes were tested in an animal model. Bone reaction was investigated at the bone-implant-interface and inside the cellular spaces of the implant. Newly formed bone was growing into the cellular spaces of the implant after 12 months. Signs of implant degradation were detected but after 12 months, no complete degradation could be observed. In conclusion, iron-based open-porous cellular biomaterials seem promising candidates for the development of self-degrading and high load bearing bone replacement materials.

## Introduction

During the last 15 years the concept of biodegradable metals has inverted the opinion regarding corrosion of temporary persistent implants. When serving as an orthopaedic implant the material ideally has to provide sufficient strength during the early stage of osteosynthesis or bone-replacement. At a later stage of bone-regeneration the degradation of the implant material introduces an augmented load transmission to the bone, and the remodelling process is supposed to be completed after a period of 6–18 months. However, due to their high stiffness massive metallic implants carry the main biomechanical load, leading to a lack of biomechanical stimulation for bone remodelling. Thus, even bone degradation may be induced by stress shielding. It has been suggested to use cellular metal materials (CMM), since those materials show Young’s moduli which are typically similar to that of cancellous bone^1–3^. Such materials offer extremely low weight and the amount of material that has to be removed by corrosion is also reduced. Open-porous metals provide access for the cells relevant for tissue reconstruction such as bone cells and blood vessels and allow their incorporation into the implant. For use as orthopaedic implants, CMM devices have been tested and commercialized during the last years on the basis of titanium and tantalum^4^.

The approach of using biodegradable magnesium with a cellular structure has been studied by Witte et al.^5^. Magnesium is highly biocompatible and features excellent osteoconductivity, but the load capacity of Mg CMM is limited and this is a significant disadvantage for a load bearing bone substitute. As alternative, iron-based alloys have been studied and initially used in cardiovascular stents^6^. Concerning the biocompatibility of iron and its alloys *in-vitro* cytotoxicity tests with MG-63 osteosarcoma cells showed that the release of iron ions leads to reduced cell proliferation rates^7^. Other groups examined the cytotoxic reactions of metal ions in bone marrow cells *in-vivo*, and showed that even the implantation of non-degradable implants resulted in higher concentrations of metal ions^8–10^. There is evidence that iron ions show cytotoxicity especially when their concentration exceeds a critical value^11^. This may explain why other authors found no toxic effects or decreased metabolic activity due to the presence of pure Fe^12,13^. Additionally, Liu et al. detected no adverse effects when various alloying elements (Mn, Co, Al, W, B, C, and S) were added^14,15^. Fagali et al. showed that the mitochondrial activity and pH-value depend on Fe^3+^ activity^16^. In vivo experiments showed that the implanted iron or tungsten stents were predominantly absorbed and did not lead to any distinct inflammatory reaction and elevated serum ion levels in blood analysis^17,18^. Other authors found limited cytocompatibility in particular of mangenese as alloying element^19–22^.

A rather low corrosion rate is a common aspect of published data on biodegradable iron and this is also reflected in the first in vivo studies using pure iron^18^. The addition of alloying elements can significantly affect the degradation rates and Hermawan *et al.* tested various Fe-Mn alloys with 20–35% manganese showing increased corrosion rates compared to pure iron^23^. Further improvements have been achieved using a Fe10Mn1Pd alloy, and Liu et al. recommended Co, W, C and S as alloying elements to increase degradation^14,24^. Our previous work showed that the addition of carbon increases the degradation rate and that small amounts of phosphorus do not entail negative effects with regards to cytotoxicity or the corrosion properties^25^. Moreover, it has been shown, that an amount of 0.6% phosphorus in powder metallurgical components has beneficial effects on the microstructure, such as sintering density and mechanical strength. This is an important finding, since the manufacturing technologies of such CMM typically are based on powder metallurgical routes^26^.

In the present paper, a Fe0.6P-alloy with two different porosities was used to manufacture cellular implants with an open-cell structure in order to test the impact of different mechanical properties on ingrowth and degradation. The implants were chemically and mechanically characterized and tested as cancellous bone replacement material in the proximal tibia of a sheep model.

## Materials and Methods

### Sample preparation

In order to produce alloys with phosphorus contents of 0.6 percent weight, carbonyl iron powder (BASF, Germany, mean particle size 4 µm) were mixed with Fe_3_P particles (Atmix, Japan, mean particle size 1.5 µm) in a ratio of 96.2: 3.8. Open-cell metal foams have been manufactured by powder metallurgical replication method. The replication method essentially involves three production steps: First a reticulated polyurethane sponge is coated by slurry impregnation. The slurry contained water, Polyvinyl-alcohol binder and the above-mentioned powder mixture with a solid content between 87 and 90%. In order to form the cellular structure reticulated polyurethane foam samples with cell sizes of 45pores per inch (ppi) (Foampartner Reisgies, Germany) were coated using double rubber rollers. Ideally, the basic pore structure of such PU foams is that of a dodecahedron as shown in earlier publications, with a diameter of approx. 1.2 mm of the large cell correlating to a cell sizes of 45 ppi^27^. The density was adjusted by controlling the coating mass of the powder suspension. This is achieved by different squeezing levels of the double rollers. Thus, it was aimed at final densities of 1.0 and 1.4 g/cm³ of the sintered components, respectively. Both implants base on the same polyurethane foam, but due to the higher coating mass, the foams with higher density shows thicker struts than those with lower density. In the next step, the components were debinded at 500 °C in ArH_2_-atmosphere and sintered at 1080 °C in pure hydrogen. Thus, open-cell foam sheets (100 × 100 × 10 mm³) were produced. In the last step, slightly conical implants (*Ø*_*bottom*_ = 10 mm, *Ø*_*top*_ = 10.2 mm, *h* = 15 mm) were cut by waterjet cutting. In order to eliminate oxides caused by the water cutting process the implants were finally reduced in pure hydrogen at 800 °C.

### Microstructure and characterization

The densities of the sintered, cut and reduced cellular structures have been measured by Archimedes method. In order to study the microstructure of the foams, metallographic cross sections were prepared and examined with optical and scanning electron microscopy (Zeiss Evo 50). The carbon content was measured using a LECO CS 230 combustion analyser. The mechanical properties were tested according to ISO 13314 by axial compressing tests using a Zwick mechanical testing machine. The crosshead speed was 0.84 mm·min^−1^ in the range up to 10% deformation and 8.4 mm·mm^−1^ at compression of >10%. Hysteresis loops as introduced by the standard have been used in order to determine the elastic properties of the material. The compressive strength was identified out of the stress strain diagrams by fitting a straight line to the linear portion of the elastic and plastic deformation, respectively, and taking the stress value at the intersection of two linear fits.

### Study design of the animal experiment

Experiments were performed according to the national guidelines for animal testing and the guidelines of the European convention for the protection of vertebrate animals for experimental and other scientific purposes. The study protocol was approved by the local animal testing board. The animal experiment was approved by the Government of Oberbayern and registered under experiment number 55.2–1–54–2531–130–07.

The statistical analysis resulted in a number of at least 11 animals per group taking an experimentally based significance level of α = 0.05 and a desired power of 0.8 as a basis. The study implemented 60 adult female merino sheep. This sort of sheep was used since it proved to be robust in previous investigations and it is frequently used in large animal models and orthopaedic experiments of this type. In the event of serious illness of an animal during the course of the experiment, replacement animals were included as was required by the animal study approval. We formed five study groups which included a control group with an identical drill hole as the one used in the other groups for implant insertion. The empty drill hole in the tibial head was marked with a K-wire at the edge to ensure identification of the region at the time of explantation. Implants of two different densities (1.0 and 1.4 g/cm^3^) were implanted and each investigated over a short term period over 6 months and a long time period over 12 months. The control animals were kept for 6 months.

We precociously included a reserve animal as soon as one of the test animals presented with pneumonia or other symptoms of an illness that would typically lead to exclusion from the experiment. Since we managed to effectively treat some animals the size of the animal test groups varied at the time of evaluation(control group n = 11, short term group 1.0 g/cm^3^ n = 11, short term group 1.4 g/cm^3^ n = 13, long term group 1.0 g/cm^3^ n = 13, long term group 1.4 g/cm^3^ n = 11).

### Surgery and anesthesiology

Preoperative pain management with meloxicam s.c. (Metacam, Boehringer Ingelheim, Switzerland) 0.5 mg/kg body weight per day was applied. Anesthesia was induced using diazepam (0.2 mg/kg body weight, Diazepam Desitin injection solution, Desitin Arzneimittel, Germany) and xylazine (0.1–0.2 mg/kg body weight, Narcoxyl injection solution, Veterinaria AG, Switzerland) and (15 mg/kg body weight, Ketavet, Pharmacia & Upjohn GmbH, Germany). Anesthesia was continued with xylazine and ketamine-hydrochloride, oxygen was delivered all along. Postoperative analgesia was performed with buprenorphine (0.324 mg, Temgesic, Fa. Essex Pharma GmbH, München, Germany). After induction of anesthesia laboratory animals were shaved at the area of operation.

**Figure 1:**
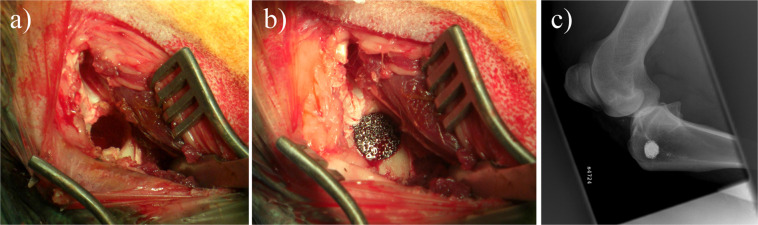
Operative site of the defect. (**a**) shows the empty drill hole, the placed implant is shown in (**b**), and (**c**) displays the implant’s location in the lateral X-ray view.

Animals were positioned in lateral decubitus position with the heads elevated in order to prevent aspiration. The surgical site was disinfected and covered with sterile sheets. A 50 mm longitudinal surgical incision was performed at the left medial tibial condyle. After dissection and preparation to bone, a 10 × 15 mm cylindrical drill hole was generated. The plasma-sterilized implant was seated into the bone defect in press-fit technique (Fig. 1).The surgical wound was closed with a suture. The procedure took approximately 30 minutes.

As perioperative prophylaxis, Augmentin 2.2 g (2.12 g amoxicillin-sodium and 238.25 mg clavulanic acid, SmithKline Beecham Pharma GmbH) was applied intravenously. Postoperative radiographic controls of the tibial condyle were performed to ensure positioning of the implants and drill holes. The animals received pain medication by means of buprenorphine and xylazine six and twelve hours after surgery. Additional pain medication was applied when animals showed signs of pain. Laboratory animals were allowed to move about freely within the corral.

Uncontrollable infection or other serious illness were defined as termination criteria for a single laboratory animal during the animal experiment. Two laboratory animals deceased during the ongoing experiment, one due to pneumonia, the other intraoperatively. Two other laboratory animals suffered from pneumonia but could be treated successfully. As a precaution two reserve animals were included in the study before the outcome of the animal treatment became obvious.

### Analysis

The experimental period ended after 6 months in the short-term group and 12 months after surgery in the long-term group and the study animals received an intravenous injection of 20–40 ml phenobarbital (Narcoren 16 g/100 ml, Merial GmbH, Hallbergmoos, Germany) for euthanasia. Dissection and tissue removal were performed subsequently. The implants were extracted with the surrounding bone tissue using a trephine (diameter of 35 mm). The material was fixed in 96% ethanol and subsequently embedded in methyl methacrylate. The orientation of the implant was identified and sections perpendicular to the long axis of the implant were obtained at a region where the implant had contact to the surrounding cancellous bone. Contact radiographs were obtained from each section and then sections were mounted on plastic slides, ground, polished and finally stained with Giemsa Eosin stain.

**Figure 2:**
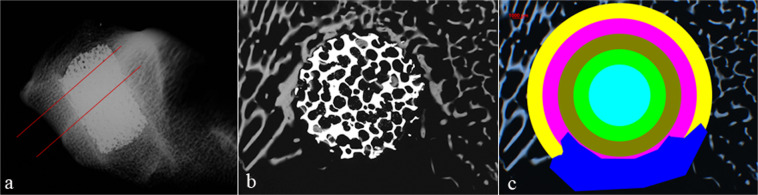
Sectional plane for contact radiography (**a**) and schematic example of contact radiography (**b**). (**c**) shows the evaluation scheme of contact radiographies with the different rings of interest (ROI) within the implant and the surrounding bone bed. The inner-most aqua ring, the lime ring and the olive ring represent the area inside the implant. The fuchsia and yellow ring represent the area of the bone bed. The dark blue area shows an area that could not be evaluated, e.g. due to contact to medullary space, therefore this area is excluded from statistical evaluation.

Histomorphometrical evaluation of contact radiographs was used to determine the amount of bone and solid implant material at certain distances from the center of the round implant cross section. For the quantitative evaluation of the bone formation inside the implants and in the bone bed, regions of interest (ROI) inside the implants‘ cross section and in the surrounding regions were defined. Measurements were conducted in 5 concentrically oriented regions, two of them lying outside the area covered by the implant cross section (Fig. 2). Implant cross sectional area was also assessed and results were compared to values of a raw and non-implanted implant.

We used semi-automated gray-scale image analysis in order to assess implant degradation and bone ingrowth. In brief, the appearing local grey levels were assigned to the implant material (appearing white in radiography), the bone (appearing at various shades of grey) and other tissues (showing no radiographic absorbance and appearing black). In order to evaluate the statistical power of the analysis various tests were carried out: The Wilcoxon-test was used to evaluate if differences in bone formation were present between groups. Furthermore, the Kruskall-Wallis-Test was performed to detect differences in bone density between the control group and the implant groups, and finally the Mann-Whitney-U-Test was used in order to assess the reactions of the same implant types with respect of the different ROI’s at different times.

At the edges of the radiographically dense metallic implant surface, a transitional change of grey levels may occur as an artefact if the implant surface is not oriented perfectly perpendicular to the plane of section. This systematic error was determined to be approximately 5.7% for both implant densities.

## Results

### Material characterization

**Figure 3:**
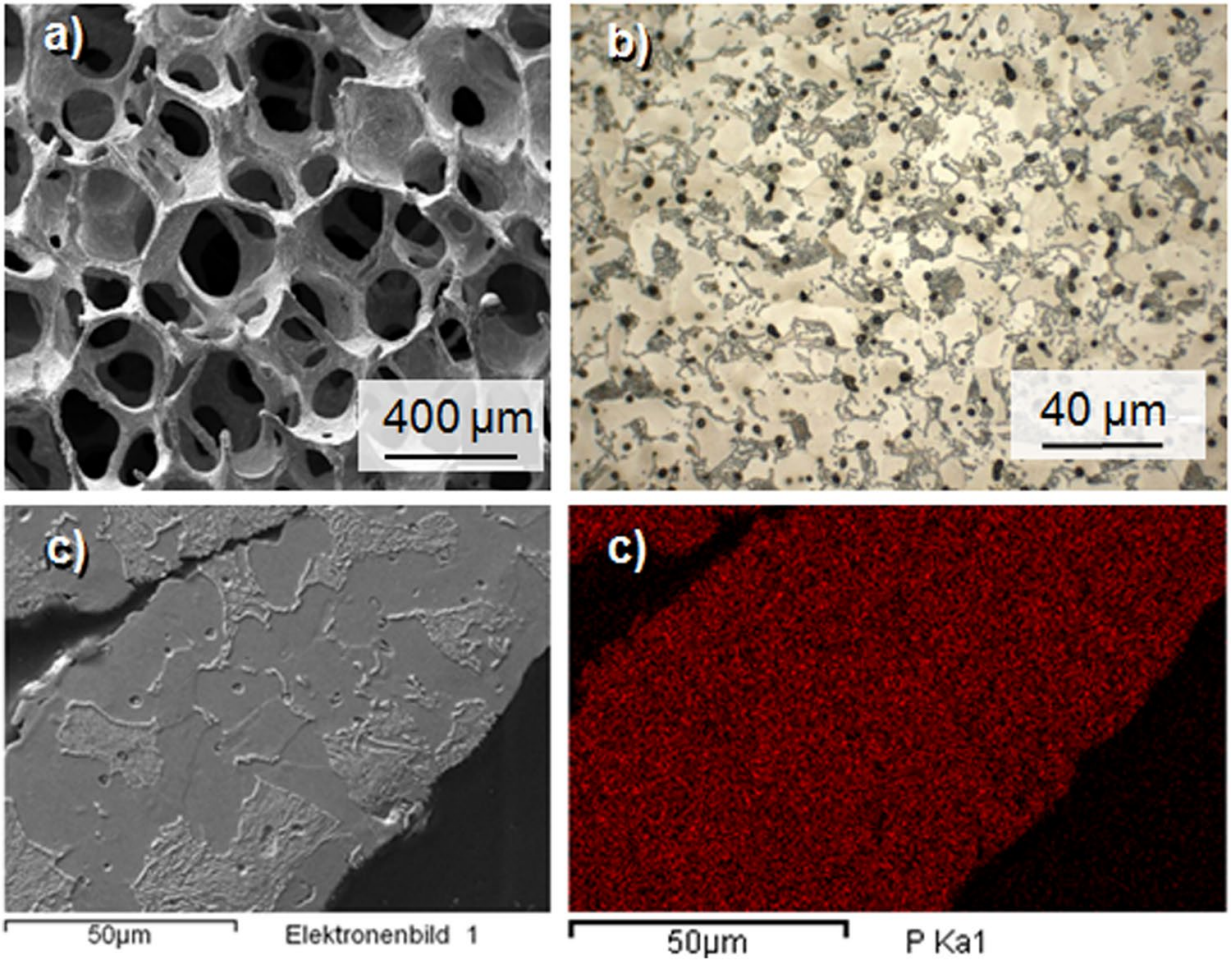
SEM-picture of an open-cell Fe0.6P structure (**a**) and light-microscopic cross section of the etched material (**b**). The microstructure displays the typical perlitic-ferritic structure of an hypoeutectoid steel. Dark dots represent micropores. SEM-picture of a cross section of an open-cell Fe0.6P structure (**c**) and EDX-Mapping of the same strut (**d**). The mapping shows a unifomly distributed phyosphorus content.

Open-cell metal implants were produced with densities of 1.0 and 1.4 ± 0.05 g/cm³, corresponding to porosities of 87 and 82 percent, respectively. The porous structure of replicated open-cell PM foams is defined by the PU foam structure with the main structural element being a pentagonal rotational ellipsoid. The latter possess cell windows which typically are 1/3 of the size of the cell itself. After heat treatment, the replicated metal foams closely resemble the original PU structure with hollow struts as a typical feature, as is displayed in Fig. 3a. However, in particular the samples with higher density reveal inhomogeneities of the mass distribution, resulting in partly closed cell windows. At a higher magnification, the microscopic cross sections show the typical pearlitic-ferritic microstructure of a hypoeutectoid steel (which is a steel with <0.8 wt.-% carbon), light grey ferritic and darker lamellar pearlite (Fig. 3b). This structure is in accordance with a measured carbon content of 0.36 wt.-% (density 1.0 g/cm³) and 0.24 wt.-% (density 1.4 g/cm³). Figure 3c shows a SEM picture of a foam strut. The same cross section is shown in Fig. 3d as an EDX-mapping of the phosphorus Kα-peak. The picture demonstrates, that the phosphorus is distributed homogenously within the strut after the heat treatment.

**Figure 4:**
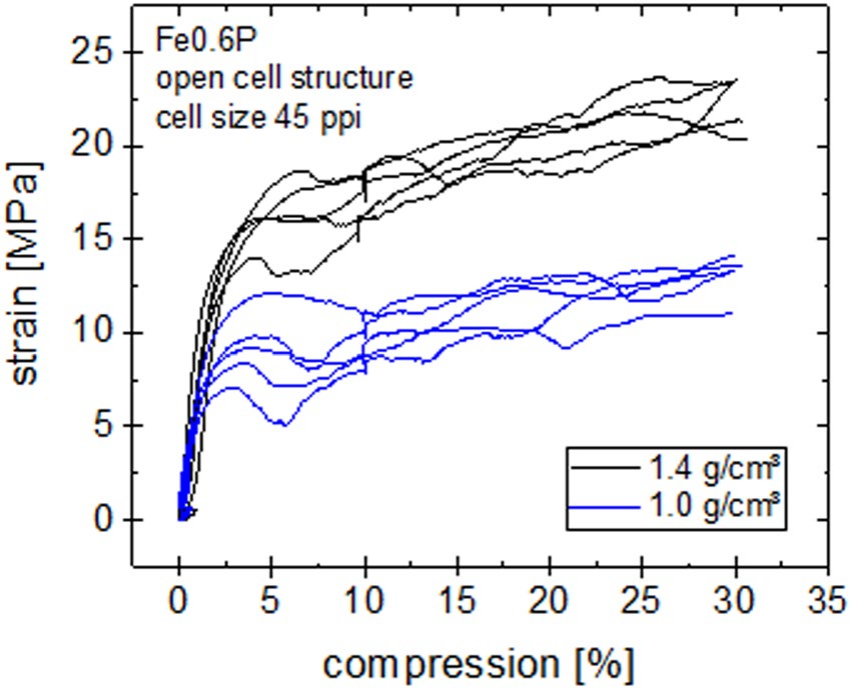
Compression test curves of the cellular Fe0.6P structures with densities of 1.0 and 1.4 g/cm³.

The mechanical properties of the implant material are displayed as scatter bands in a stress-strain diagram (Fig. 4). These curves exhibit the typical behaviour of cellular metals with a small region of elastic deformation followed by a plateau region, where changes in compression do not change the stress explicitly. The compressive strength was determined by fitting a straight line to the linear portion of the elastic and plastic deformation, respectively, and taking the stress value at the intersection of these 2 lines. Thus, compressive strengths of 13.1 MPa and 22.8 MPa, respectively, were measured, and Young’s moduli of 0.8 and 1.3, respectively, were detremined (see Table 1).

**Table 1:** Carbon content, oxygen content and compression strength of implanted open-cell structures.

density [g/cm³]	carboncontent [wt.-%]	oxygencontent [wt.-%]	compressionstrength [MPa]	youngsmodulus [GPa]
1.0	0.36 ± 0.03	0.06 ± 0.04	13.1 ± 1.2	0.8 ± 0.2
1.4	0.24 ± 0.11	0.05 ± 0.03	22.8 ± 1.1	1.3 ± 0.2

### Histopathological characterisation

Sections were qualitatively evaluated and the amount of bone surrounding the implant and invading the implant was recorded. In addition the amount of implant material which appeared black in transmitted light mode (and white in contact radiography) was assessed.

**Figure 5:**
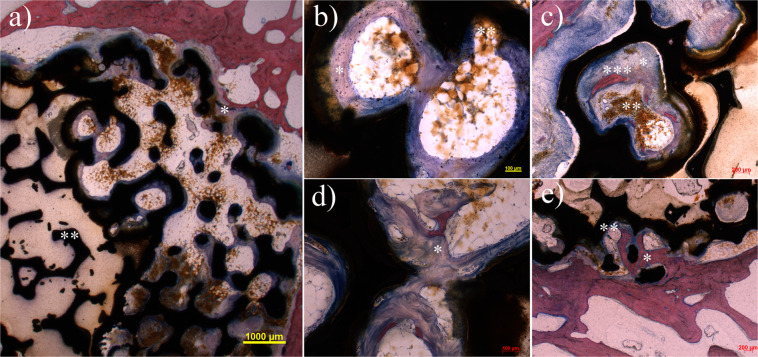
Overview of a cross section 6 months after operation with standard Giemsa-Eosin staining shows the beginning ingrowth (*) of the implant (**a**). Giant cells are found at the interface at the edge of the implant as sign of absorption of corrosion particles. The central area includes an implant area with closed pores (**). Such regions appear in a few of the implants. Biological activity cannot be detected in this specific area in contrast to the open pore areas. After 6 months the 1.0 g/cm^3^ group (**b**) and the 1.4 g/cm^3^ group (**c**) show distinct formation of osteoid at the implant (*) and degradation of implant particles (**), in particular at the bone-implant interface. Individual areas marginally already show slight mineralization (***). After 12 months the 1.0 g/cm^3^ group (**d**) exhibit progressive formation of osteoid with formation of a trabecular network (*). Mineralization of the newly built bone has not yet taken place. After the same period the 1.4 g/cm^3^ group after shows extensive mineralization of the newly built osteoid at the margin contacting the implant (*). The immediate bone-implant interface shows a thin osteoid margin that is not yet mineralized (**).

In all types of implants regions with direct contact between newly formed bone and implant surface were observed (Fig. 5).The outer diameter of the porous implant was clearly recognizable in all specimens. There was no sign of structural reduction of the implant material with the exception of a brown to yellow appearing material which could be observed within the implant cavities and in the immediate neighbourhood. In some cases the material was present in the mononuclear cells which fills the spaces between bone and implant surface. In particular at the later time points larger amounts of solid particles were detached from the implant and multinuclear macrophages were detected inside the implant. A local inflammatory reaction in terms of leucocytosis or lymphocytosis was not detectable.

#### Control group

After 6 months, the bone defects of the control group were completely filled with newly formed bone tissue and could only be identified using the implanted k-wire. The structure of the bone within the region of the former drill hole did not differ from normal bone tissue with respect to osteoid formation.

#### Group 1.0 g/cm^3^

Short-term group.—One implant had to be excluded from evaluation due to dislocation into the bone marrow. In the remaining implants newly formed osteoid was present in the bone tissue outside and inside the implant. The osteoid appeared blue in Giemsa Eosin stain and this usually identifies newly formed bone tissue with very low mineral content. In one animal beginning mineralisation of the newly formed tissue could be identified by red stained regions within the blue appearing osteoid. Five animals showed distinct formation of new bone at the implant-bone interface. Four animals showed a modest formation of new bone at the implant-bone interface. One animal did not show any signs of new bone tissue formation at the implant-bone interface. Signs of severe local inflammation or rejection of the implant were not observed although multinuclear cells were occasionally present.

Long-term group. In all animals non-mineralized osteoid formation was detected, which was pronounced less in two cases. Seven of the animals showed significant bone formation surrounding the implant.

#### Group 1.4 g/cm^3^

Short-term group. One implant had to be excluded due to secondary dislocation and was excluded from evaluation. In all other animals, non-mineralized osteoid was demonstrated within regions lying inside the porous implant. Non-mineralized osteoid was also present at the implant-bone interface in 12 out of 13 cases.

Long-term group. In 10 of 11 animals newly formed and centrally mineralized osteoid was found inside the implant. The implant-bone interface is covered to a large extent with non-mineralized osteoid. In the immediate neighbourhood to the implant newly formed and mineralized bone was detected in all cases.

### Quantitative analysis of contact radiography

**Figure 6:**
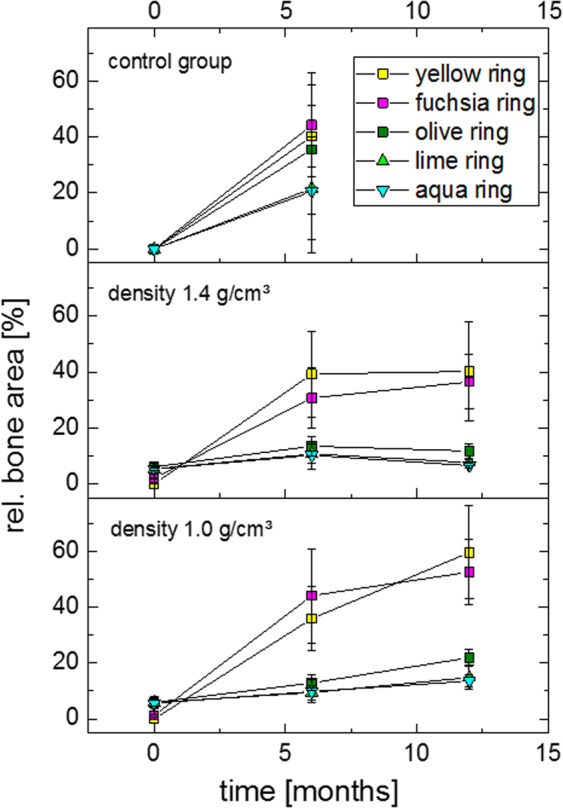
Newly build bone formation shown in contact radiography in a follow up of 12 months in the group of 1.0 g/cm^3^ (below) and 1.4 g/cm^3^ (middle) and the control group (above).

By analysing the contact radiographs, quantitative data on the newly built bone has been generated. Figure 6 shows the relative area of bone formation of the groups of implant density of 1.0 and 1.4 g/cm³ as well as the control group with respect to the different ROIs. In detail, the following results are shown:

- Both groups show a significant bone formation in the outermost area of the bone bed after 6 months. After 12 months, a significant increase of newly built bone for the 1.0 g/cm^3^ group (p < 0.05) is shown, but not for the 1.4 g/cm^3^ group.
- At the bone bed area adjacent to the implant (shown in fuchsia), no significant differences between time points 6 and 12 months were found for both implant types. The outermost area inside the implant shown as olive ring showed a significant difference in formation of new bone in the group of 1.0 g/cm^3^ over time (p < 0.05), but not for the group of 1.4 g/cm^3^.
- Inside the implant at the medium ring (lime colour), a significant increase of bone density over time (p < 0.05) was detected in the group of 1.0 g/cm^3^. In contrast, the group of 1.4 g/cm^3^ showed a significant decrease of bone density between the 6^th^ and 12^th^ month (p < 0.05). Finally, the centre of the implant (aqua ring) reveals a significant increase of bone density analogously to the lime ring (p < 0.05) in group 1.0 g/cm^3^ over time. In group 1.4 g/cm^3^ bone density was significantly lower (p < 0.05) in the long-term group compared to the short-term group

In order to verify the explanatory power of the results, statistical tests were performed. In this context, the Wilcoxon-test gives an information about the difference of new formed bone in relation to the baseline including the partial volume effects of the implants. Thus it reveals a significant difference between the bone formation in inner regions of all implant groups and the raw implant. Furthermore, the Kruskall-Wallis-Test was performed in order to detect differences in bone density between the control group and the implant groups. Thus, in the surrounding regions of the implant no differences appeared. However, in both implant groups the bone density at the inner implant region was significantly lower compared to the control group. Finally, reactions at different time points of the same type of implant were assessed using the Mann-Whitney-U-Test for each of the different ROI’s.

### Implant degradation

**Figure 7:**
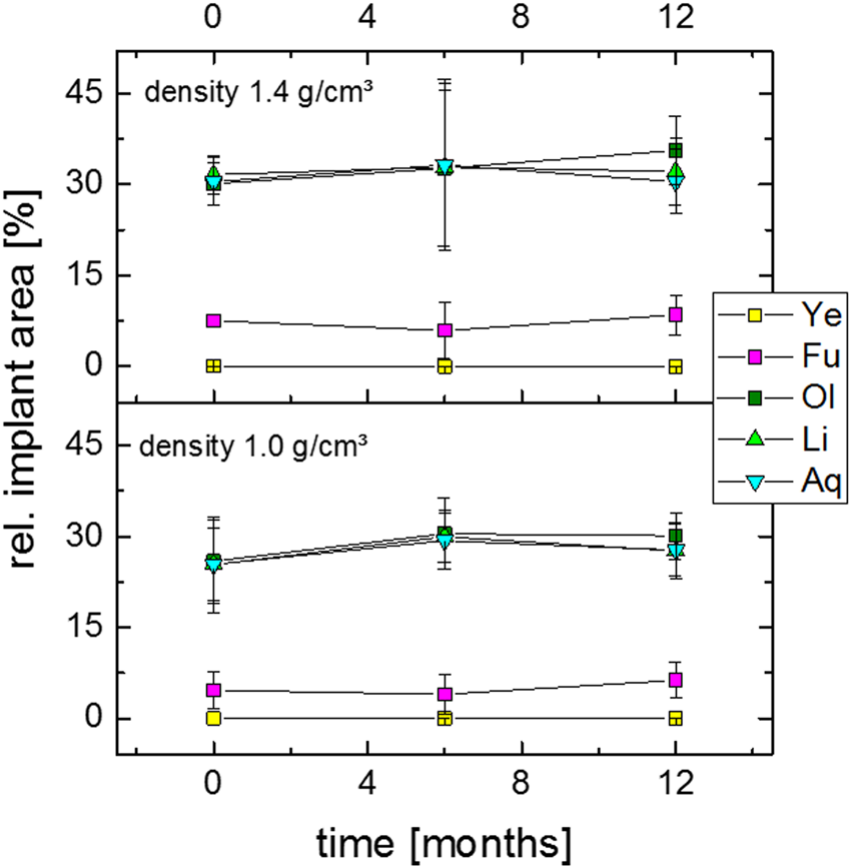
Relative area of the open-cell implant shown in contact radiography in a follow up of 12 months in the group of 1.0 g/cm^3^ (above) and 1.4 g/cm^3^ (below).

The quantitative analysis of the relative cellular implant area as given by the contact radiographs is shown in Fig. 7. At the inner rings (aqua, lime, olive), the data shows no significant change within the duration of the study. This finding is even shown by the Mann-Whitney-U-Test. These findings are independent of the implant density and the point of time.However, the histological cross sections revealed degradation products to a relevant degree and some particles, which had been detached from the implant. Furthermore, phagocyted particles in macrophages which are in touch with the implant have been detected.

## Discussion

In the present study cellular metals have been manufactured out of an iron alloy for the use as a bone replacement material which has shown biodegradable properties in vitro. The cellular material structure closely resembles the structure of spongy bone, and due to its distinctive roughness it is easy to fix in the bone bed and thus provides a good primary stability. An essential property is the mechanical strength, which should be comparable to the bony strength. The ultimate strength of the presently tested material is comparable to human cancellous bone (0.9–14 MPa)^28^, but clearly lower than cortical bone (97–183 MPa)^29–31^. As shown by Gibson and Ashby, an increase in strength of cellular metals can be achieved by increased structural density or by increasing the matrix material strength^32^. In the present case, phosphorus has been added as an alloying element due to its known solution hardening effect^26^. Furthermore, as indicated by the Fe-P phase diagram small amounts of 0.6 wt.-% phosphorus are dissolved in the iron matrix and lead to the presence of a γ-phase at sintering temperature (1080 °C). Since the diffusion coefficient of the γ-phase is increased by factor ~100 phosphorus in small amounts leads to an increased sintering activity and thus decreases the microporosity of the cell struts^33^.

The Young’s modulus of such materials is also a function of structural density, enabling an adjusting of a mismatch between the bony modulus and the implant. Tibial bones typically show moduli between 0.02 and 0.5 GPa, thus the stiffness of the cellular iron implants used in the present study is only slightly higher^28^. Such tailoring of implant stiffness has a significant effect when using bioinert permanent materials like titanium or tantalum, but also in the present work neoformation of bone has been detected, mainly at the edges of the implant^4^. Even in the center of the implants, newly formed bone was identified. However, iron-based alloys feature reduced cell proliferation characteristics^7,25^, thus the kinetic of the ingrowth is reduced compared to the control group or spongy titanium, which shows exceptional osteoconductivity^34^. On the other hand, in accordance to the results of Peuster *et al.*^18^, we saw no inflammatory reactions either after 6 months or after 12 months. This is due to the slow release of degradation particles. A faster release is likely to result in more pronounced inflammation. In conclusion, the newly developed material reveals adequate biocompatibility. Anyway, due to the reduced rate of bone ingrowth the adaption of the implant stiffness to the bony environment is of increased relevance.

In the present work, the density of the metallic cellular implants was relatively high. As a result, CMM produced by powder metallurgic replication method may show reduced openness of the pores^26^. As a consequence, distinctive areas occur, where closed pores cause screening effects and prohibit further bony integration. Beyond that, the effects of reduced stiffness in the 1.0 g/cm³ group are rather low. Indeed, the groups with different implant porosities exhibit slightly different ingrowth capabilities in particular in the second period of the test after 12 months. But anyway, the statistic variations of the metal cellular structures with different openness of the pores appear to have the more pronounced effect. This is reflected notably in the strong fluctuation range of the mechanical strength. A significant increase of the degradation rate seems to be achievable by targeted material design as shown by several authors

An important finding of the present work is the rather low degradation rate of the iron basis implants. This behavior is in contrast to earlier *in vitro* experiments. Several authors report controllable corrosion rates which have been achieved by means of alloying or microstructural manipulation^14,19,21,24,35^ and moreover, an appropriate corrosion behavior of the same alloy as used in the present study has been determined *in vitro* with approximately 0.26 mg/cm^2^∗d.^26^ Despite the fact that the internal surface of the implants in the present work has been quite high due to their cellular structure on the other hand, the *in vivo* experiments reveal more or less intact implants. The same findings have been reported by Kraus et al. after in-vivo tests of bulky Fe21Mn0.7C1Pd implants which have been tested for 12 months^36^. Nevertheless, initial degradation is visible in the form of degradation products. Obviously, these degradation products form passivation layers on the implant surface, whose dissolution behavior in the bony environment is quite low and not reproducible in the in vitro experiment. However, the histological analysis suggests, that degradation products are removed in the form of isolated particles are by phagocytes, but the rate of this removal process is not quantifiable. Consequently, the degradation is reduced to a minimum after the formation of a thin passivation layer. The particles shown are most likely real degradation particles. Wear particles would require mechanical stress. This is unlikely because of the press-fit fixation in the bone. Furthermore, we were able to show in histological images and contact recordings that an ingrowth of the bone has taken place, which speaks for a stable fixation. These findings are in accordance with earlier in vivo experiments with pure iron stents^18^ or with Fe30Mn alloys^23^.

With regard to the reduced degradation the presented data suggests only a limited suitability of the implant concept, despite of the uncritical cytotoxicity of the tested cellular Fe0.6P material. Nevertheless, the reduction in implant mass by using cellular metals with high porosities and high strength seems to be reasonable when alternative basic materials are targeted. Thus, the strength could be kept at the level of spongy bone and a further reduction of the implant stiffness and lowered stress for the organism could be achieved^26^.

## Conclusion

With this study it is demonstrated that open porous implants on the basis of Fe0.8P show good biocompatibility. Bone regenerates and grows into the implant´s pores showing well-mineralized bone tissue at the interface between bone and implant after 12 months. However, the implants degrade quite slowly. Further development of the alloy as well as of the structure of the implant will be needed to promote faster ingrowth and regeneration of mineralized bone. The rate of degradation should be increased significantly. A completely open-porous structure has to be provided since occluded caverns cannot be penetrated by newly built bone. We set the base for the further development of a completely new degradable and fully weight-bearing biomaterial which can provide primary stability to large bone defects after implantation and features slow degradation.
